# Circulating microRNAs and prediction of asthma exacerbation in childhood asthma

**DOI:** 10.1186/s12931-018-0828-6

**Published:** 2018-06-26

**Authors:** Alvin T. Kho, Michael J. McGeachie, Kip G. Moore, Jody M. Sylvia, Scott T. Weiss, Kelan G. Tantisira

**Affiliations:** 1 Channing Division of Network Medicine, Brigham and Women’s Hospital, 181 Longwood Avenue, Boston, MA 02115 USA; 2 Harvard Medical School, 25 Shattuck Street, Boston, MA 02115 USA; 3 Computational Health Informatics Program, Boston Children’s Hospital, 320 Longwood Avenue, Boston, MA 02115 USA

**Keywords:** Asthma exacerbation, Circulating microRNA, Biomarker

## Abstract

**Background:**

Circulating microRNAs have shown promise as non-invasive biomarkers and predictors of disease activity. Prior asthma studies using clinical, biochemical and genomic data have not shown excellent prediction of exacerbation. We hypothesized that a panel of circulating microRNAs in a pediatric asthma cohort combined with an exacerbation clinical score might predict exacerbation better than the latter alone.

**Methods:**

Serum samples from 153 children at randomization in the Childhood Asthma Management Program were profiled for 754 microRNAs. Data dichotomized for asthma exacerbation one year after randomization to inhaled corticosteroid treatment were used for binary logistic regression with miRNA expressions and exacerbation clinical score.

**Results:**

12 of 125 well-detected circulating microRNAs had significant odd ratios for exacerbation with miR-206 being most significant. Each doubling of expression of the 12 microRNA corresponded to a 25–67% increase in exacerbation risk. Stepwise logistic regression yielded a 3-microRNA model (miR-146b, miR-206 and miR-720) that, combined with the exacerbation clinical score, had excellent predictive power with a 0.81 AUROC. These 3 microRNAs were involved in NF-kβ and GSK3/AKT pathways.

**Conclusions:**

This combined circulating microRNA-clinical score model predicted exacerbation in asthmatic subjects on inhaled corticosteroids better than each constituent feature alone.

**Trial registration:**

ClinicalTrials.gov Identifier: NCT00000575.

**Electronic supplementary material:**

The online version of this article (10.1186/s12931-018-0828-6) contains supplementary material, which is available to authorized users.

## Background

Asthma is a chronic inflammatory respiratory disease characterized by airway obstruction due to both smooth muscle hyperresponsiveness and inflammation [1]. Both asthma therapies and hospitalizations/doctor visits generate significant healthcare utilization [2]. Specifically, an estimated US$62.8 billion was spent on the diagnosis and management of asthma in the U.S. in 2009 with inflation adjusted costs continuing to rise [3]. Asthma is the leading chronic disease cause of hospitalizations and school absences in children [4]. Individuals with frequent asthma exacerbations represent those with the greatest morbidity and economic cost due to asthma [5, 6]. Thus, identification of children at highest risk for exacerbations could result in personalized care and substantially improve outcomes. The current guidelines that determine asthma control are related to clinical symptoms, lung function, and recent history of exacerbations [7]. Despite ongoing efforts in asthma management and control, acute exacerbations continue to be a significant health care problem. Numerous studies have attempted to predict asthma exacerbations in children using a variety of clinical and biomarker inputs. These include clinical prediction scores and exhaled breath condensate interleukin-5 level [8], fractional exhaled nitric oxide and inflammatory markers in exhaled breath condensate alone and combined with clinical variables [9], recent severe asthma exacerbations as an independent predictor of future severe exacerbations [10], and addition of single nucleotide polymorphisms (SNPs) in concert with clinical parameters to predict exacerbation outcomes [11]. In the latter, the authors used genome-wide genotypic data (~ 550,000 SNPs) to determine children at risk for a severe asthma exacerbation. A predictive panel consisting of 417 children and a validation sample of 164 children was used; however, the addition of 160 SNPs to clinical data only increased the model Area Under the Receiver Operator Characteristic (AUROC) curve from 0.54 to 0.66 showing good, but not excellent, predictive power. Similarly, each of the other aforementioned models has had variable success with respect to prediction of asthma exacerbation [12], and there is ongoing need for improved prediction of asthma clinical outcomes and discovery of biomarkers that achieve this goal.

MicroRNAs (miRNA) are short, single-stranded RNA molecules approximately 19–23 nucleotides that regulate gene expression with various functions described in the literature [13]. Circulating miRNAs are attractive biomarkers for prediction of disease outcomes [14] and are stable in the serum over long time frames even in harsh conditions [15]. Circulating miRNA has been studied as a non-invasive biomarker in heart transplant rejection prediction [16], prediction of metastasis in breast cancer patients with early disease [17], and prediction of thiazolidinedione response in diabetes prevention [18]. Prior studies of miRNA in asthma have focused mainly on asthma case-control status, including studies involving circulating miRNA expression in childhood asthmatics compared to healthy controls [19], regulation of IL-5 expression by miRNA differential expression in serum of asthmatics and health controls [20], and differential expression of miRNA in epithelial and airway cells [21]. A recent study showed that a subset of circulating miRNAs is expressed uniquely between asthma and allergic rhinitis patients [22]. However, prediction of important clinical events, such as asthma exacerbations, using circulating miRNAs has not been performed.

Our study investigated asthma exacerbation prediction in the 12 months following randomization to the inhaled corticosteroid treatment arm in 153 subjects in Childhood Asthma Management Program (CAMP) [23]. The miRNA model was compared to a clinical score of exacerbation risk [24]. Subsequently, we assessed the use of a combined miRNA and exacerbation clinical score model. We hypothesized that a miRNA model or a combined miRNA-clinical score model would have predictive capabilities superior to the clinical score alone. We identified a panel of 3 miRNAs with good predictive power comparable to the clinical score. One of the 3 miRNAs targets genes is well known in asthma pathobiology, while the others have been associated with asthma. The combined miRNA-clinical score model showed very good predictive power superior to either the miRNA or clinical score models alone.

## Methods

### CAMP inclusion criteria and definition of asthma exacerbation

The CAMP study was a multi-center, randomized, double-blinded clinical trial that investigated the safety and efficacy of inhaled budesonide versus nedocromil versus placebo in 1041 pediatric patients over a mean follow-up of 4.3 years. The CAMP Genetics Ancillary Study was approved by the Brigham and Women’s Hospital Internal Review Board, protocol # 2015P001622/BWH. Informed consent and assent was obtained from parents and participants respectively.

The trial has been described [25]. Briefly, the inclusion criteria were for 5–12 year old children at time of screening, with chronic asthma symptoms for at least 6 months in the year prior to interview, PC_20_ < 12.5 mg/mL, and other factors. Children were excluded if their asthma was severe, had a confounding or complicating condition, or the child could not perform acceptable spirometry or methacholine challenge. Diary card and other clinical characteristics were collected prior to randomization.

Asthma exacerbations were defined as the need for oral prednisone for treatment of asthma which are also called steroid bursts [23]. Clinics instructed patients/caregivers to recognize an asthma exacerbation based on symptoms or by decrease in peak flow to < 80% personal best. Oral prednisone was prescribed “if the patient uses more than 12 puffs of albuterol in 24 hours”, or had symptom code described as “one or more asthma episodes that last longer than 2 hours or result in shortened normal activity, seeing a doctor for acute care, or going to a hospital for acute care”, or if peak flow dropped to < 50% of personal best despite bronchodilator usage. Prednisone dose and duration in addition to tapering protocol were specified in the protocol, and the physician could decide on an extended course of oral steroid depending on the clinical response.

### miRNA profiling

miRNA profiling of serum from 160 CAMP subjects taken at the start of CAMP (i.e., enrollment or randomization to treatment) using TaqMAN miRNA quantitative PCR primers (Life Technologies Megaplex RT Primers, Human Pool Set v3.0, Omaha, Nebraska) containing 754 primers representing 738 unique human miRNAs (miRBase release 14) was previously described [26]. The initial quality control was performed per manufacturer protocol using pre-defined thresholds for amplification scores (> 1.24) and Cq (> 0.80) confidence intervals. All miRNA in this paper are annotated using miRBase release 14 [27] (http://www.mirbase.org/). The complete miRNA dataset is accessible at the NCBI Gene Expression Omnibus (GEO, https://www.ncbi.nlm.nih.gov/geo/) GSE74770.

Analysis of biological replicates was performed in 10% of the samples showing high miRNA-miRNA correlations (rank correlations of > 0.90; data not shown). All subjects were self-identified non-Hispanic Caucasians to limit the effect of race on miRNA expression [28], and subjects were selected from the inhaled corticosteroid arm of the trial. For data analysis, quantile normalization on the detected miRNAs was performed sample-wise to the mean of the data matrix using MatLab (MathWorks Inc., Natick, Massachusetts) function *quantilenorm*. A miRNA was included in the analysis if expression was present in at least 70% of subjects: 125 miRNAs passed this selection criterion. 7 subjects were excluded due to incomplete steroid burst usage data yielding 153 subjects for analysis. For subjects with replicate miRNA sample profiles, only replicates #2 were used in this analysis.

### Data analyses.

#### Asthma exacerbation clinical score calculation

Using a previously described approach for calculating asthma exacerbation risk [24], a clinical score for predicting pediatric asthma exacerbation was calculated from the baseline history performed during CAMP enrollment in addition to parental survey data [24]. The scoring table is reproduced in Additional file 1: e-Table S1. Since, leukotriene modifiers were not used in clinical practice at time of study, we capped the total maximum score from the clinical score to 15 (rather than total of 16).

#### Logistic regression models

Data for asthma exacerbation from the first year after randomization to the inhaled corticosteroid arm was used. Consistent with the National Asthma Education and Prevention Program (NAEPP) guidelines [29], patient data were dichotomized into exacerbation status with a total steroid burst counts of 0–1 (status 0, no exacerbation) and > 1 (status 1, exacerbation). Logistic regression was performed using R with this binary exacerbation status as the dependent variable, and miRNA cycle threshold (CT) values or exacerbation clinical score as the independent variable, separately [30]. Both univariate and multivariate models adjusting for clinical co-variates (age, sex) were performed. Missing miRNA CT values were replaced by the sample-wise median in order to perform backwards stepwise selection on significant miRNAs and clinical variables with *p*-value < 0.05. Goodness of fit for each model was assessed with the Hosmer-Lemeshow test in the R package *ResourceSelection* [31]. The likelihood ratio test was performed comparing the different models with R package *lmtest* [32].

#### Model validation, receiver operator characteristic (ROC) curves and area under ROC curves

R package *caret* [33] was used to create a 60% training and 40% testing set balancing the class distributions of the outcome, steroid bursts. The R function *predict* was used to predict the outcomes of the models on the testing data set. ROC curves and Area Under the ROC curve (AUROC) were calculated using R packages *pROC* [34] and *ROCR* [35]. R package *cvAUROC* [36] was used to perform 10-fold cross validation of AUROCs on the full data set.

#### Identification of miRNA-gene targets and pathway analysis

miRTarBase [37] Release 6.0 was used to identify gene targets of the 3 miRNAs (miR-146b, miR-206 and miR-720) with “Functional MTI” support type, i.e., functionally validated targets. Pathway analysis with the gene list was performed with use of Database for Annotation, Visualization and Integrated Discovery (DAVID version 6.8, https://david.ncifcrf.gov//) [38].

## Results

### Study population

Population characteristics of the 153 CAMP subjects stratified by asthma exacerbation status are shown in Table 1**.** 38 of 153 (25%) of subjects experienced an exacerbation during the first year of the trial, despite being randomized to inhaled corticosteroids. The cohort was restricted to self-identified non-Hispanic whites to eliminate confounding from race on miRNA expression [28].

**Table 1:** Study cohort characteristics at the start of CAMP (except # of steroid bursts in one year) stratified by asthma exacerbation status

No. Subjects	No Exacerbation^a^ *N* = 115	Exacerbation^a^ *N* = 38	
Characteristic	Mean (SD)	Mean (SD)	*p*-value
Age – years	8.9 (2.0)	8.9 (2.2)	0.93
Male sex – %	54%	61%	0.48*
Height – centimetre	133.2 (13.4)	132.8 (13.8)	0.86
Body Mass Index – percentile	61.5 (28.3)	63.1 (26.4)	0.75
Forced Expiratory Volume pre-bronchodilator – litre	1.7 (0.5)	1.6 (0.5)	0.71
# of steroid bursts in one year^a^	0.3 (0.4)	3.2 (1.7)	1.4 × 10^− 12^
Clinical Asthma Exacerbation Score (# of subjects)	Low 13 Average 43 High 59	Low 1 Average 10 High 27	0.08**^**

(*SD*) is standard deviation, where present

^a^Exacerbation status: Participant who received 0–1 steroid burst in one year were classified as “no exacerbations.” Two or more steroid bursts were classified as “exacerbation”

*Chi-square test. **^**Fisher’s exact test. Others *p*-values calculated by two-sided t-test assuming non-equal variance

### miR association and prediction of asthma exacerbation

**Fig. 1:**
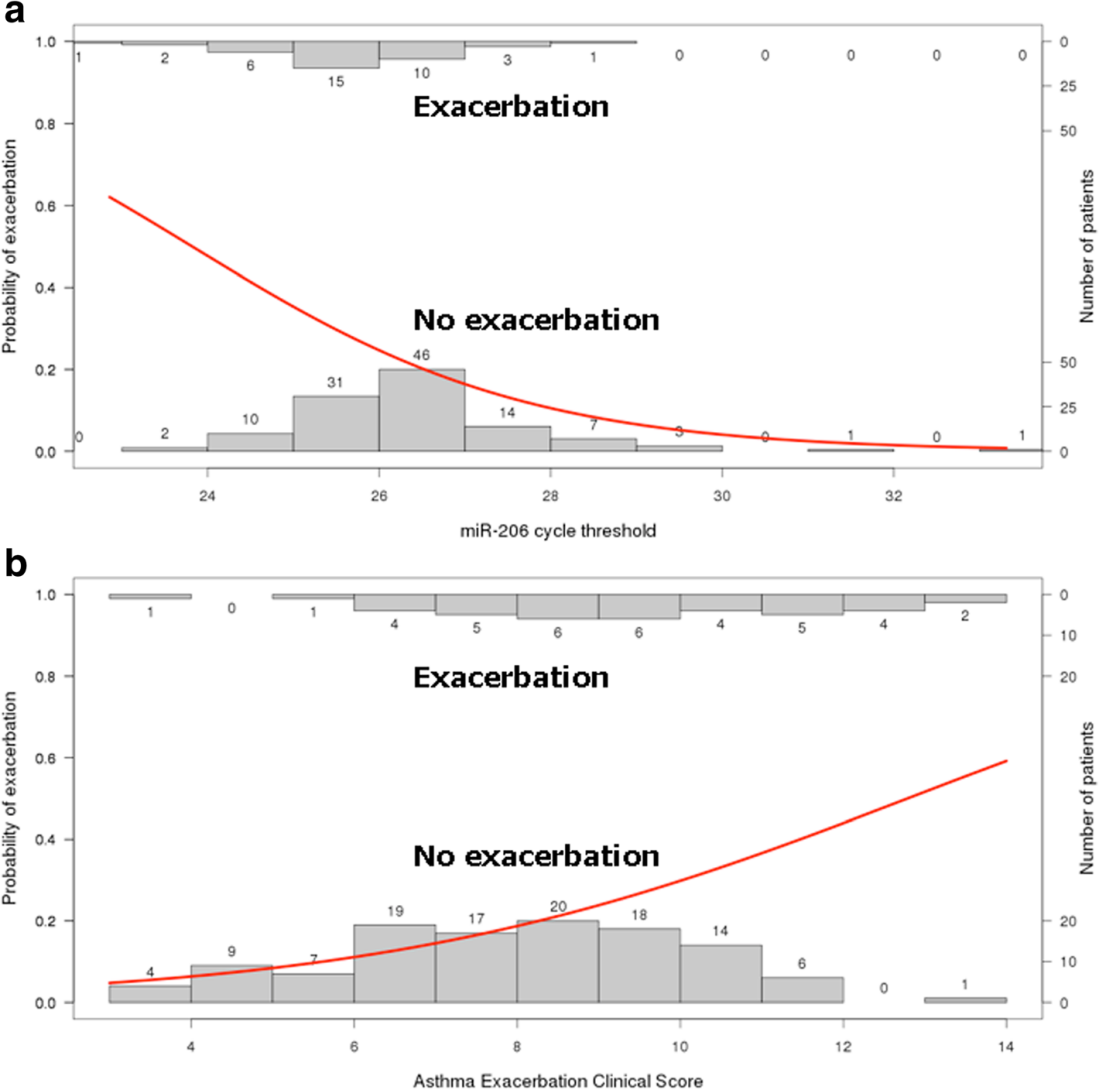
Univariate logistic regression models for miR-206 expression (Panel **a**) and asthma exacerbation clinical score (Panel **b**) relative to exacerbation. The horizontal axis represents the miRNA cycle threshold and asthma exacerbation clinical score for panels **a** and **b**, respectively. The right vertical axis represents number of patients. In each panel, the top (inverted) histogram represents subjects who had an exacerbation and the bottom histogram represents subjects who did not have an exacerbation. The red line represents the unadjusted logistic regression function with probability of exacerbation on the left vertical axis. For instance in panel **a**, as the miR-206 cycle threshold increases (abundance in blood decreases), the risk of asthma exacerbation decreases. Whereas in panel **b**, as the asthma exacerbation clinical score increases, the risk of asthma exacerbation increases

Logistic regression showed 12 of the 125 interrogated miRNA were associated with exacerbation for pediatric asthma exacerbation within the first year following randomization (Table 2). The strongest miRNA association for asthma exacerbation was miR-206 with a logistic regression odds ratio (OR) of 0.60 (95% CI: 0.42–0.83). The miR-206 (Panel A) and exacerbation clinical score (Panel B) logistic functions are shown in Fig. 1. All 12 miRNA associations had OR < 1 indicating decreasing risk of asthma exacerbation with increasing miRNA CT (i.e. decreasing abundance of circulating miRNA). Overall, each doubling of expression of the 12 associated miRNAs was associated with a 25 to 67% (corresponding to OR 0.8 to 0.6) increase in risk of exacerbations. Following our initial univariate models, we formulated a prediction model from the 12 associated miRNAs using backwards stepwise logistic regression. Following selection, a 3-miRNA model showed best fit – miR-146b-5p, miR-206 and miR-720 (Table 3). Interestingly, we had previously reported 3 of the 12 (miR-223-5p, miR-339-3p, miR-454-3p) to be associated with baseline FVC%, and 6 of the 12 (miR-126-3p, miR-146b-5p, miR-206, miR-342-3p, miR-409-3p, miR-454-3p) to be associated with baseline FEV1/FVC [26].

**Table 2:** Univariate (unadjusted) logistic regression model for microRNAs relative to exacerbation with missing cycle threshold values replaced by its sample-wise cycle threshold value median

microRNA	Odds ratio (OR)	95% Confidence Interval (CI)	*p*-value
miR-206^c^	0.60	0.42–0.83	0.004
miR-146b-5p^c^	0.66	0.48–0.89	0.007
miR-222-3p	0.70	0.52–0.93	0.02
miR-409-3p^c^	0.73	0.56–0.95	0.02
miR-223-5p^b^	0.62	0.40-0.92	0.02
miR-126-5p^c^	0.68	0.48–0.93	0.03
miR-339-3p^b^	0.72	0.53-0.96	0.03
miR-30e-3p	0.70	0.49–0.95	0.03
miR-126-3p	0.74	0.56–0.96	0.03
miR-342-3p^c^	0.80	0.64–0.98	0.04
miR-454-3p^a^	0.77	0.60-0.98	0.04
miR-720	0.71	0.50–0.98	0.046

^a^miRNA previously reported to be associated with baseline ^b^FVC% and baseline ^c^FEV1/FVC [26]

**Table 3:** Summary of logistic regression models relative to exacerbation with coefficient odds ratios, 95% confidence intervals and other model measures. Each column represents the regression coefficients for each of the 3 models

Variable	miR model^a^	Clinical model	miR + Clinical model
hsa-miR-206^b^	0.64**^$^ (0.45,0.89)	__	0.65** (0.44, 0.92)
hsa-miR-146b	0.72** (0.52,0.98)	__	0.67** (0.47,0.93)
hsa-miR-720	0.75 (0.51, 1.1)	__	0.70* (0.46,1.03)
Clinical score	__	1.36*** (1.14,1.64)	1.38*** (1.15,1.69)
Age	__	1.01 (0.84,1.23)	0.97 (0.80,1.20)
Sex	__	0.79 (0.36,1.73)	0.70 (0.29,1.65)
Observations	153	153	153
Log Likelihood	−76.8	−79.5	−69.9
Akaike Information Criterion (AIC)	161.6	167	153.7
Hosmer-Lemeshow Goodness of Fit Test**^**	Χ^2^ = 9.15 p-value = 0.33	Χ^2^ = 8.45 p-value = 0.39	Χ^2^ = 7.07 p-value = 0.53

^a^The miRNA model was determined by backwards stepwise selection. The clinical score model was adjusted for age and sex per original publication

^b^Each of the model coefficients presents the odds ratio and the 95% confidence interval in parenthesis

*P*-values are marked by an asterisk (*) with: *p < 0.1, **p < 0.05, ***p < 0.01

**^**10 bins used to calculate quantiles with degree of freedom = 8

### miR vs. clinical score models

The logistic regression model for exacerbation status relative to the exacerbation clinical score had an OR 1.36 (95% CI: 1.14–1.64) indicating an increasing risk of asthma exacerbation with increasing clinical score. The miRNA, clinical score and combined (miRNA-clinical score) models are summarized in Table 3**.** Likelihood ratio testing indicated that training set combined (miR-clinical score) model was superior to the clinical model alone (Χ^2^ = 11.7, *P*-value 0.009).

**Fig. 2:**
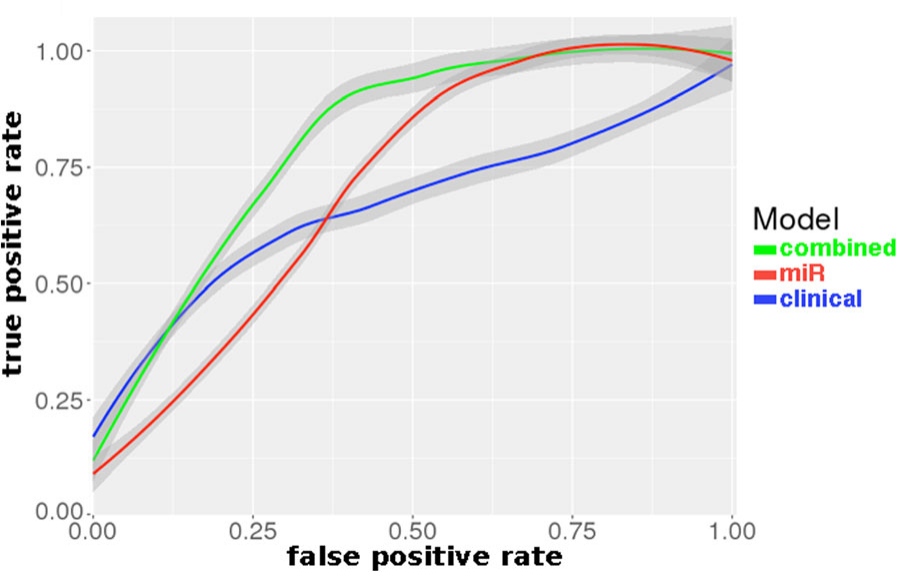
Comparison of Receiver Operator Characteristic curves between 3 models. Asthma exacerbation clinical score (blue), miRNA (red) and combined (miRNA and clinical score, green) models in the testing set data. AUROCs were 0.671, 0.714, and 0.807, respectively. A linear LOESS smoothing function was applied with 95% confidence interval shown

Model prediction was assessed via ROC curves generated for the miRNA, clinical score and combined models testing set data (Fig. 2). The combined model showed good predictive power (AUROC 0.81), significantly better than the clinical model (AUROC 0.67) or miRNA model (AUROC 0.71). 10-fold cross-validation for AUROC was performed on the full data set showing that the estimated AUROC for the combined model retained good predictive value and remained higher than both miRNA and clinical model (Additional file 1: e-Table S2)**.**

### Gene ontology and pathway analysis

Next we assessed miRTarBase-identified functionally-validated gene targets of the 3 miRNAs (miR-146b, miR-206 and miR-720) for pathway and ontologic enrichment. 2 of the 4 top significant pathways in DAVID Biocarta Pathway Analysis were asthma-related: “Inactivation of GSK3 by AKT causes accumulation of b-catenin in alveolar macrophages” (FDR *p*-value 1.7 × 10^− 2^) and “NF-κβ signaling pathway” (FDR p-value 8.0 × 10^− 2^) (Additional file 1: e-Table S3). Multiple gene targets of these miRNAs were in the “Inactivation of GSK3 by AKT causes accumulation of b-catenin in alveolar macrophages pathway” (Additional file 1: e-Figure S1). The most significantly enriched DAVID Gene Ontology category was “positive regulation of fibroblast proliferation” (FDR p-value 1.5 × 10^− 2^) (Additional file 1: e-Table S4).

## Discussion

In this study, we examined baseline circulating miRNA in childhood asthmatics prior to treatment with inhaled corticosteroids (ICS) to predict exacerbations in the subsequent year. We noted that 12 miRNAs (Table 2) were significantly associated with future exacerbations, with each doubling of expression of these miRNAs associated with a 25–67% increase in risk of exacerbations. We had previously reported 3 of the 12 (miR-223-5p, miR-339-3p, miR-454-3p) to be associated with baseline FVC%, and 6 of the 12 (miR-126-3p, miR-146b-5p, miR-206, miR-342-3p, miR-409-3p, miR-454-3p) to be associated with baseline FEV1/FVC [26]. When combined, 3 miRNAs (miR-146b-5p, miR-206 and miR-720) by themselves provided comparable predictive power to an established clinical model of exacerbations. Moreover, when these 3 miRNAs were combined with the clinical factors included in the model, there was significant increase in the ability to predict future exacerbations with AUROC 0.81.

Notably, all of the subjects within the current study were randomized to inhaled corticosteroids as part of a clinical trial cohort. Therefore, while our findings may apply broadly to asthma exacerbations as a whole, this information may also have pharmacogenomic implications via the identification of subjects who experience exacerbations despite therapy with inhaled corticosteroids. Therefore, validation of these findings may identify subjects who may benefit from alternate, or additional, therapies.

Our top miRNA, hsa-miR-206, was significantly associated with subsequent asthma exacerbations with OR 0.60 (95% CI: 0.42–0.83) (Table 2). Given that the distribution of expression for miR-206 is broad (spanning multiple cycle thresholds, Fig. 1, Panel A), this suggests that the reported OR for the individual miRNAs are likely conservative. Figure 1 shows the difference between the miRNA (Panel A) and asthma exacerbation clinical score (Panel B) logistic regression functions with fairly dispersed values for exacerbation status compared to a division at miR-206 cycle threshold around 26. This suggests miR-206 discriminates exacerbation status better than clinical score.

Stepwise selection of significant miRNAs resulted in a 3-miRNA model (miR-146b, miR-206 and miR-720) that was subsequently compared to and combined with an asthma exacerbation clinical score model (Table 3**)**. The combined miR-clinical score model had very good predictive power to discriminate exacerbation from no exacerbation (AUROC 0.81). This AUROC is higher than prior clinical studies focused solely on the asthma exacerbation clinical score [24] with an AUROC of 0.75 for a Costa Rican cohort and AUROC 0.69 for the CAMP cohort and Childhood Asthma Control Test (C-ACT) with AUROC 0.72 [39]. The AUROC is also improved compared to other biomarkers including addition of our prior study of GWAS SNPs from the same cohort [11]. The combined miR-clinical score model far exceeded the predictive capability for exacerbation status compared to the miRNA or clinical score model alone. These data support the hypothesis that optimal prediction models for personalized medicine are likely to come from a combination of ‘ omics and clinical variables.

The miRNAs used to build our predictive model overlaps with miRNAs relevant to asthma pathobiology. miR-146b, along with miR-146a, are negative regulators of inflammatory gene expression in lung tissue [40]. A murine model study of acute and chronic asthma showed consistent upregulation of miR-146b, which is also expressed by leukocytes and has been shown as a negative regulator NF-κβ in human breast cancer cells [41]. This could potentially explain why circulating miR-146b may be a viable biomarker. In eosinophilic esophagitis, miR-146b has been found both in esophageal biopsies and differentially regulated in the plasma [42]. Altered expression in the airway wall of the other 2 miRNAs in our predictive model, miR-206 and miR-720, were noted in a mouse model of childhood allergic asthma [43]. Moreover, miR-206 has been shown to be involved in airway smooth muscle (ASM) innervation [44], thereby enhancing the mechanistic significance of our model. Integrative miRNA studies would be needed for direct elucidation of interactions of our miRNAs as they relate to asthma pathobiology. Pathway analysis (Additional file 1: e-Table S3) also had borderline significance for the NF-κβ pathway, which correlates to the aforementioned miR-146b regulatory role. Genes affected by the miRNA were also involved in the inactivation of GSK3 by AKT causes accumulation of b-catenin alveolar macrophages pathway (Additional file 1: e-Figure S1 and e-Table S3). Inactivation of GSK3 has been studied in a murine model and is associated with ASM hypertrophy and possibly linked to asthmatic airway remodeling [45]. Overall, further study of these miRNA may suggest a functional approach to small RNA-directed therapies to treat or prevent asthma exacerbations.

This study has several strengths including a large number of interrogated miRNAs, large sample size of pediatric asthma patients from the CAMP cohort, biologically significant miRNAs found in modeling, and comparison to a prior validated asthma exacerbation clinical score. The CAMP cohort is clinically well-characterized notably for both identification and protocol-based treatment of asthma exacerbations, which should reduce measurement error. Replicate analysis discussed in the methods section showed high miRNA-miRNA correlations. While the primary CAMP trial recruitment occurred several years ago, circulating miRNAs have been shown to be stable over the course of many years [46, 47]. While we do not yet have independent replication, the maintenance of high AUROCs in our cross-validation analyses supports potential generalizability.

## Conclusions

From a survey of 738 baseline circulating miRNA in childhood asthmatics prior to ICS treatment, we identified 12 miRNAs that were significantly associated with exacerbations in the subsequent year, with each doubling of expression of these miRNAs associated with a 25–67% increase in risk of exacerbations. When combined, 3 miRNAs (miR-146b-5p, miR-206 and miR-720) by themselves provided comparable predictive power to an established clinical model of exacerbations. Moreover, when these 3 miRNAs were combined with the clinical factors included in the model, there was significant increase in the ability to predict future exacerbations with AUROC 0.81. To our knowledge, this is the first study to investigate prediction of asthma exacerbations with miRNAs.

Our results are promising for the translation of circulating miRNA to predict clinical outcomes related to asthma and are consistent with prior studies using circulating miRNA as biomarkers, predictors, or markers of treatment response. miRNA as potential biomarkers for a priori prediction of asthma exacerbations may be particularly salient, given the substantial morbidity and health care costs associated with exacerbations. Additionally, since this particular study restricted subjects to inhaled corticosteroid therapy, its findings may have direct pharmacogenomic relevance in the prediction of which participants respond to ICS therapy. Further study of miRNA alone or in concert with other genomic and epigenomic markers may reveal additional predictive power for asthma risk assessment and even treatment responses.

## Additional file

Additional file 1: Circulating MicroRNAs and Prediction of Asthma Exacerbation in Childhood Asthma. **e-Table S1.** Asthma Exacerbation Clinical Score. **e-Table S2.** Cross-validation (10-fold) of AUROC (all data). **e-Table S3.** DAVID Biocarta Pathway Analysis. **e-Table S4.** Top DAVID GOTERM_BP DIRECT. **e-Figure S1.** DAVID (Database from Annotation, Visualization, and Integrated Discovery) Biocarta Pathway analysis - Inactivation of GSK3 by AKT causes accumulation of b-catenin in alveolar macrophages. miRTarBase 6.0 was used to determine experimentally validated microRNA-target interactions with genes. The gene list was subsequently used for pathway analysis. The genes marked with the red star are targeted by the microRNA. (DOCX 956 kb)

## Abbreviations

95% CI: 95% confidence interval
AUROC: Area under the Receiver Operator Characteristic curve
CAMP: Childhood Asthma Management Program
CT: Quantitative real time polymerase chain reaction cycle threshold
FDR: False discovery rate
ICS: Inhaled corticosteroids
miRNA: microRNA
OR: Odds ratio
ROC: Receiver Operator Characteristic curve
SNP: Single nucleotide polymorphism
