# Supplementary material for: Circulating microRNAs and prediction of asthma exacerbation in childhood asthma
Source: Respir Res. 2018 Jun 26;19:128. doi: 10.1186/s12931-018-0828-6 (PMC6020199; doi:10.1186/s12931-018-0828-6)
Supplement: Supplementary file 1 — Circulating MicroRNAs and Prediction of Asthma Exacerbation in Childhood Asthma. e-Table S1. Asthma Exacerbation Clinical Score. e-Table S2. Cross-validation (10-fold) of AUROC (all data). e-Table S3. DAVID Biocarta Pathway Analysis. e-Table S4. Top DAVID GOTERM_BP DIRECT. e-Figure S1. DAVID (Database from Annotation, Visualization, and Integrated Discovery) Biocarta Pathway analysis - Inactivation of GSK3 by AKT causes accumulation of b-catenin in alveolar macrophages. miRTarBase 6.0 was used to determine experimentally validated microRNA-target interactions with genes. The gene list was subsequently used for pathway analysis. The genes marked with the red star are targeted by the microRNA. (DOCX 956 kb) [file 12931_2018_828_MOESM1_ESM.docx]

## Title: Circulating MicroRNAs and Prediction of Asthma Exacerbation in Childhood Asthma

## ONLINE SUPPLEMENTARY MATERIALS

|  |  |
| --- | --- |

## e-Table 1: Asthma Exacerbation Clinical Score

# Reported asthma symptoms

1. Symptoms for >= 3 months of the year (1 point)

Question 81 on Baseline Asthma and Allergy History. If symptoms were present for >= 3 months, one point awarded.

1. Symptoms precipitated by colds, cold air, exercise, dust (1 point each)

Each Variable (Question 43, 46, 39, and 36, respectively) was coded from 0-3 with 0 meaning “never causes asthma” to 3 “always or almost always causes asthma. One point was awarded if the answer was 1,2, or 3.

# Current Asthma Medications

1. Short-acting B2 agonist (1 point each)

Question 24: Variable was coded from 1-5 with 4-5 meaning less than once per week and never, respectively. No point was awarded if scored as 4 or 5.

1. Inhaled steroids (1 point each)

Question 20: In the last 6 months how often has your child used ICS for asthma? As with question 24, no point was awarded if scored as 4 or 5 (less than once per week and never, respectively)

1. Leukotriene inhibitors (1 point each)

The CAMP trial did not use LTM.

# Healthcare utilization

1. Ever hospitalized for asthma (1 point)

Question 16: If the answer was (yes/1), 1 point awarded.

1. Ever admitted to ICU for asthma (1 point)

Question 20: If the answer was (yes/1), 1 point awarded.

1. = 2 courses of steroids last year (1 point)

Question 30: Referred to >= 2 courses of steroid in 6 months rather than 1 year. Most subjects did not require steroid therapy. If the answer was >=2, 1 point was awarded.

1. = 2 ER visits for asthma last year

Question 21: If there was >=2 ER visits in a year, 1 point was awarded

1. Doctor visits last year - 3 or more (1 point)

Question 22

1. Doctor visits last year - 6 or more (1 point)

Question 22

# Medical History

1. Personal history of eczema/hay fever (1 point)

Questions 92 and 96

1. Parental history of asthma or atopy (1 point)

Derived from parental history data – father and mother self-reported status was utilized

1. Smoke exposure as infant or current (1 point)

Derived from parental history data – father and mother self-reported status was utilized

# Total Score

1. Low risk (score ≤ 5)
2. Intermediate or average risk (score 6-8)
3. High risk (score ≥ 9)

**e-Table 2: Cross-validation (10-fold) of AUROC (all data)**

|  | miR-clinical model | miR model | Clinical model |
| --- | --- | --- | --- |
| cvAUROC | 0·74 | 0·66 | 0·66 |
| 95 % CI | 0·66 – 0·82 | 0·57 – 0·75 | 0·56 – 0·75 |

**e-Table 3: DAVID Biocarta Pathway Analysis**

| Pathway | Gene Count | P-value | Corrected P-value^ |
| --- | --- | --- | --- |
| Inactivation of Gsk3 by AKT causes accumulation of b-catenin in Alveolar Macrophages | 5 | 2·4x10^-4^ | 1·7x10^-2^ |
| Influence of Ras and Rho proteins on G1 to S Transition | 4 | 3·9x10^-3^ | 6·5x10^-2^ |
| Cyclins and Cell Cycle Regulation | 4 | 3·1x10^-3^ | 8·0x10^-2^ |
| NF-κβ Signaling Pathway | 4 | 2·3x10^-3^ | 8·0x10^-2^ |

^ Benjamini-Hochberg correction

**e-Table 4: Top DAVID GOTERM_BP DIRECT**

| Term | Gene Count | Percentage of Genes | P-value | Corrected P-value^ |
| --- | --- | --- | --- | --- |
| Positive regulation of fibroblast proliferation | 4 | 12·9 | 1·2x10^-4^ | 1·5x10^-2^ |
| Positive regulation of phospholipase C activity | 3 | 9·7 | 1·1x10^-4^ | 1·8x10^-2^ |
| Pentose-phosphate shunt | 3 | 9·7 | 1·7x10^-4^ | 1·8x10^-2^ |
| Positive Regulation of gene expression | 6 | 19·4 | 9·2x10^-5^ | 2·0x10^-2^ |
| Negative regulation of transcription from RNA polymerase II promoter | 8 | 25·8 | 2·2x10^-4^ | 2·0x10^-2^ |

^ Benjamini-Hochberg correction


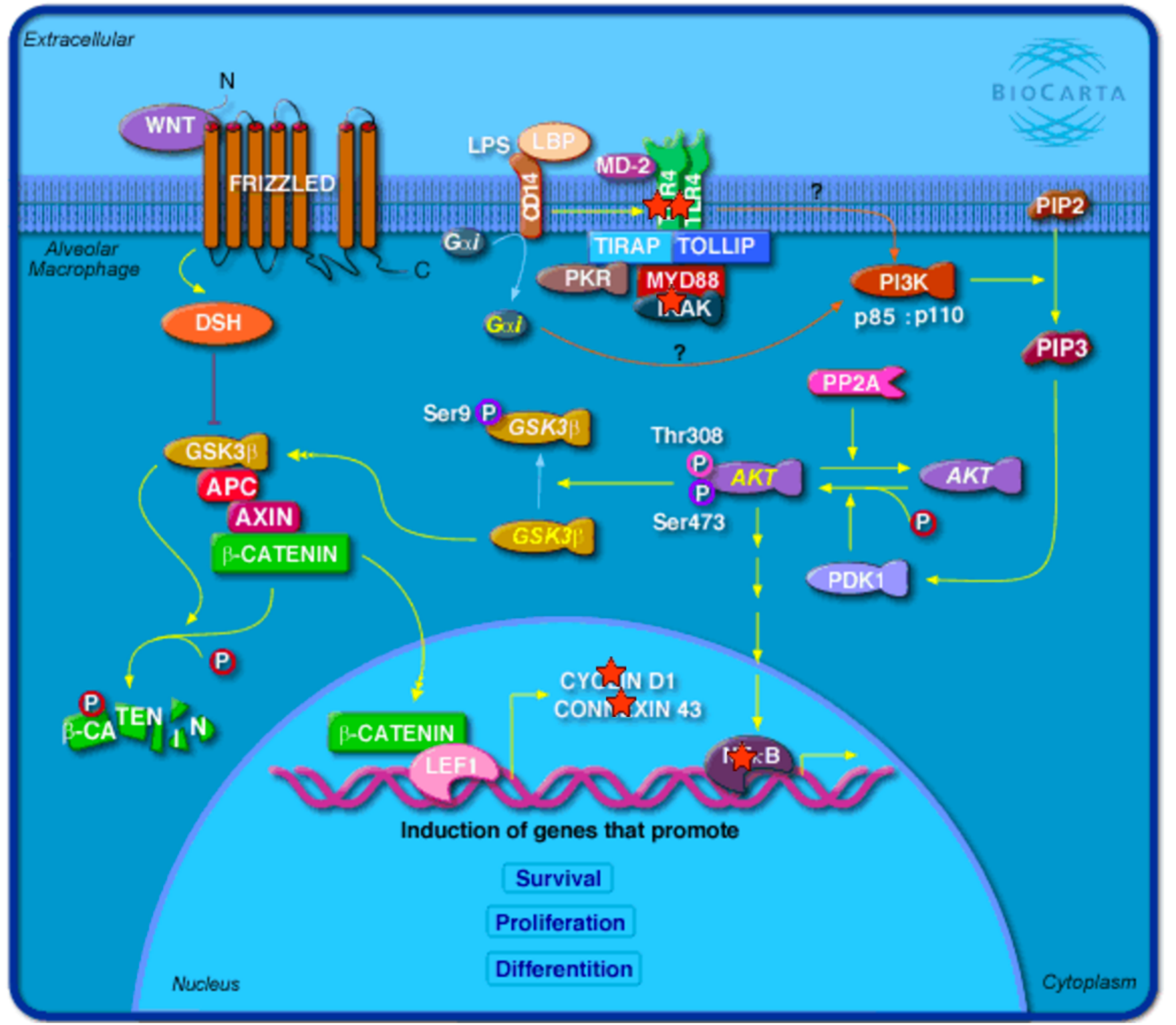


**e-Figure 1**: DAVID (Database from Annotation, Visualization, and Integrated Discovery) Biocarta Pathway analysis - Inactivation of GSK3 by AKT causes accumulation of b-catenin in alveolar macrophages. miRTarBase 6.0 was used to determine experimentally validated microRNA-target interactions with genes. The gene list was subsequently used for pathway analysis. The genes marked with the red star are targeted by the microRNA.

|  |  |  |
| --- | --- | --- |

**References**

1. The Childhood Asthma Management Program (CAMP): design, rationale, and methods. Childhood Asthma Management Program Research Group. Controlled clinical trials. 1999;20(1):91-120.
